# Understanding of mouse and human bladder at single‐cell resolution: integrated analysis of trajectory and cell‐cell interactive networks based on multiple scRNA‐seq datasets

**DOI:** 10.1111/cpr.13170

**Published:** 2021-12-23

**Authors:** Bowen Shi, Yanyuan Wu, Haojie Chen, Jie Ding, Jun Qi

**Affiliations:** ^1^ Department of Urology School of Medicine Xinhua Hospital Affiliated to Shanghai Jiao Tong University Shanghai China

**Keywords:** bladder, cell‐cell communication, scRNA‐seq, trajectory analysis

## Abstract

**Objectives:**

To elaborately decipher the mouse and human bladders at single‐cell levels.

**Materials and Methods:**

We collected more than 50,000 cells from multiple datasets and created, up to date, the largest integrated bladder datasets. Pseudotime trajectory of urothelium and interstitial cells, as well as dynamic cell‐cell interactions, was investigated. Biological activity scores and different roles of signaling pathways between certain cell clusters were also identified.

**Results:**

The glucose score was significantly high in most urothelial cells, while the score of H3 acetylation was roughly equally distributed across all cell types. Several genes via a pseudotime pattern in mouse (Car3, Dkk2, Tnc, etc.) and human (FBLN1, S100A10, etc.) were discovered. S100A6, TMSB4X, and typical uroplakin genes seemed as shared pseudotime genes for urothelial cells in both human and mouse datasets. In combinational mouse (n = 16,688) and human (n = 22,080) bladders, we verified 1,330 and 1,449 interactive ligand‐receptor pairs, respectively. The distinct incoming and outgoing signaling was significantly associated with specific cell types. Collagen was the strongest signal from fibroblasts to urothelial basal cells in mouse, while laminin pathway for urothelial basal cells to smooth muscle cells (SMCs) in human. Fibronectin 1 pathway was intensely sent by myofibroblasts, received by urothelial cells, and almost exclusively mediated by SMCs in mouse bladder. Interestingly, the cell cluster of SMCs 2 was the dominant sender and mediator for Notch signaling in the human bladder, while SMCs 1 was not. The expression of integrin superfamily (the most common communicative pairs) was depicted, and their co‐expression patterns were located in certain cell types (eg, Itgb1 and Itgb4 in mouse and human basal cells).

**Conclusions:**

This study provides a complete interpretation of the normal bladder at single‐cell levels, offering an in‐depth resource and foundation for future research.

## Graphical abstract

Multiple scRNA‐seq datasets of normal mouse and human bladder were collected. Through standard workflow of Seurat R package, we identified cell clusters in bladder tissue. After initial exploration of differentially expressed gene and gene set enrichment analysis, we performed trajectory analysis for pan‐fibroblasts and urothelial cells and identified common pseudotime genes across datasets and species. Then, we integrated multiple datasets to create, to date, the largest cell map of mouse and human bladder, respectively. We further explored the details of signaling pathways between certain cell types and the communication networks of specific pathways.

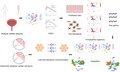

## 1 INTRODUCTION

Single‐cell sequencing provides unprecedented resolution to help us understand and analyze the process of normal tissue growth and disease occurrence.^1^ In particular, intercellular communication analysis at the single‐cell level plays an essential role in helping us analyze physiological and pathological states.^2^ The bladder is an important organ in the human urinary system, and the comprehensive analysis at the single‐cell level is helpful for us to elaborate and explore its regular physiological basis and also in order to establish the foundation for further dissecting of certain changes in conditions of inflammation or tumor. Previous large‐scale cell atlas analysis^3^, ^4^, ^5^ has constructed the fundamental structs of multiple organs, but none of them comprehensively decomposed the details of the bladder. On the contrary, scattered data from prior reports^6^, ^7^ associated with bladder (single‐cell level) with a limited number of cells per study may not be able to provide an all‐round understanding. Yu et al.^8^ have reported an admirable study sketching a single‐cell transcriptomic map of bladder in both mouse and human, but this research is entirely lacking any cell‐cell interaction analysis. Li et al.^9^ have demonstrated a novel cell type labeled by Plxna4 using scRNA‐seq in the mouse bladder, but this study is only focusing on the urothelial layer and without cell communicative network analysis or validation of any human samples. Studies have suggested that cross talk between cells profoundly impacts the development and the regeneration of the respiratory system.^10^ Data from scRNA‐seq also provide insights into the landscape of intercellular cross talk of liver cells in health and disease.^11^ On the basis of multiple datasets, we comprehensively analyzed the bladder data of human and mice at the single‐cell level and focused on the trajectory analysis of urothelial and interstitial cells in the bladder and their interactions between cells.

## 2 METHODS

### 2.1 Single dataset preprocessing and normal bladder at the single‐cell level

First, to establish initial independent analysis datasets for mouse and human bladder, respectively, we collected single‐cell RNA sequencing data of normal bladder tissues from GSM4201633^12^ (mouse, 3′ 10X Genomics, Illumina HiSeq 2500) and GSM4850577^6^ (human, 5′ 10× Genomics, HiSeq X Ten). Then, data were analyzed using Seurat (v3.0 and v4.0)^13^ in R (version 4.0.0) following standard workflow from website vignette (https://satijalab.org/seurat/). Uniform manifold approximation and projection for dimension reduction (UMAP) was done as the preferable way to display the most clustering data unless it is not available. After clustering of cells, the identification of cell groups was primarily according to the previous well‐known marker genes^7^, ^14^ using the “FindAllMarkers” function which identifies differentially expressed genes between one cluster and all other cells using Wilcoxon rank‐sum tests.

The cell‐cycle score was calculated by the “CellCycleScoring” function which would assign each cell a score, based on its expression of G2/M and S‐phase markers, and other scores (eg, hypoxia, metabolism, and histone modifications) were added by the “AddModuleScore” function which could calculate supervised module scores for any given gene list. R Package clusterProfiler^15^ (version 3.18.1) was used for gene set enrichment analysis (GSEA) using differentially expressed genes (both positive and negative) across cell clusters. Most GSEA gene lists were obtained from the msigdbr (version 7.1.1) package. Genes associated with N^6^‐methyladenosine (M6A) modifications were manually gathered from the published articles.^16^, ^17^ Developmental trajectory and pseudotime analysis of pan‐fibroblasts in mouse and human bladder was performed using monocle3^18^ (version 0.2.1). Of note, these two datasets were considered independent analysis objects and were not into subsequent integration.

### 2.2 Multiple datasets preprocessing and independent trajectory analysis of urothelium and pan‐fibroblasts in bladder

In addition to the above two datasets, single‐cell transcriptomic data of normal bladder tissues from GSE129845^8^ (three patients and two mice, 3′ 10X Genomics, HiSeq X Ten Illumina), GSE134355^5^ (two patients, microwell‐seq, HiSeq X Ten Illumina), GSE108097^4^ (one mouse, microwell‐seq, Illumina HiSeq 2500), GSE109774^3^ (one mouse, smart‐seq2 and 3’10x Genomics, Illumina NovaSeq 6000) were further collected. All expression data were filtered (quality control of doublet, low‐quality cells or empty droplets, and extensive mitochondrial or ribosomal contamination) in R (version 4.0.0) using Seurat (v3.0 and v4.0) package.

Our initial preliminary attempts found that the integration process would affect the subsequent trajectory analysis of urothelial and interstitial cells in the bladder. Whether reciprocal principal component analysis (RPCA) or canonical correlation analysis (CCA), both would impair the significance of typical pseudotime genes. So, we performed trajectory analysis on each sample (with the available amount of cells), respectively, using the monocle3 package. Top genes (with the highest Moran I score) were displayed in scatter plots. In addition, the top 50 genes of each analysis via a pseudotime pattern were collected, and then mitochondrial or ribosomal genes were excluded; the selection range would expand to the top 60–100 if there were not sufficient left genes (n > 25) after removal. These genes were showed in Venn plots to explore the potential common key trajectory genes by intersection.

### 2.3 Integrated mouse and human bladder datasets and cell‐cell interactions

Then, these multiple datasets were integrated into the mouse and human combined datasets, respectively, based on the “FindIntegrationAnchors” function, which can find a set of anchors between a list of Seurat objects, and these anchors can later be used to integrate the objects with CCA method. Then, these cells were subsequently normalized, scaled, linear‐dimensional/nonlinear reduced, and clustered by Seurat mainly using default parameters.^13^ Cell‐type identities were assigned on the basis of the present cluster biomarkers (differentially expressed gene) and the previously reported marker genes of human and mouse bladder.^8^ Cell‐cell communication networks were identified and visualized by CellChat (Version 1.0.0) package.^19^ Nebulosa (v1.3.0) R package was used to calculate and visualize the joint density of co‐expression patterns.

## 3 RESULTS

### 3.1 Overview of the single dataset of mouse and human bladder at the single‐cell level

**FIGURE 1:**
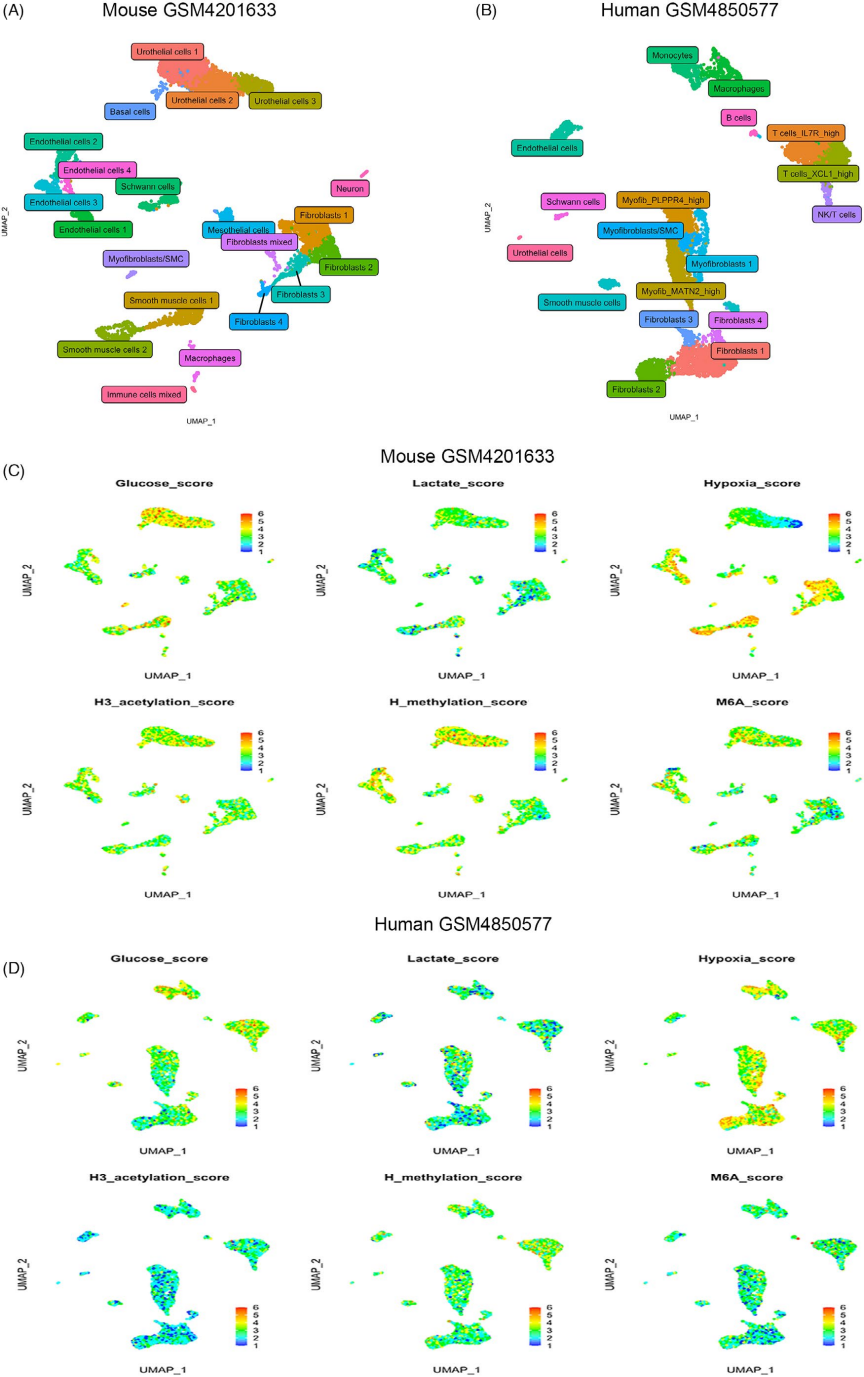
UMAP plots and biological activity score of cell clusters in initial two datasets. (A) Identified cell types in mouse bladder. (B) Identified cell types in human bladder. (C) Module scores of glucose and lactate metabolism, hypoxia, methylation, acetylation, and M6A in mouse bladder. (D) Module scores of glucose and lactate metabolism, hypoxia, methylation, acetylation, and M6A in human bladder. M6A: N6‐methyladenosine. Abbreviations: H, histone; H3, histone 3

After quality control, 6,962 and 6,982 cells, respectively, of mouse and human bladder were analyzed. In mouse and human bladder, we recognized 21 and 18 cell clusters, respectively (Figure 1A‐B). Cell identity was almost consistent with previous and their source papers. But, we reconsidered the definition of a particular cluster described as fibroblast/smooth muscle cell (SMC) by Shuai He et al.^6^ Most fibroblast/SMC were identified as myofibroblasts in our study, which is more often used and widely accepted in other researches. Only a few cells with extremely high SMC markers (eg, ACTA2, ACTG2, and CNN1) and relatively low typical fibroblast markers (eg, S100A4, COL3A1, and COL1A1) were defined as the myofibroblasts/SMC cluster. Besides, the human bladder dataset (GSM4850577) was lacking in urothelial cells, and it might be caused by different sampling procedures compared with other studies.^6^, ^8^ Though the mouse bladder dataset (GSM4201633) contained a pool of eight mice bladders, it seemingly had a relatively low cell capture per bladder which might be due to its stricter dissection and recombination processing for samples.^12^

### 3.2 Marker gene of cell clusters in mouse and human bladder

For both human and mouse bladder, the following markers collected from the previous reports were applied for clusters' identification.^3^, ^4^, ^6^, ^8^, ^12^, ^14^ Keratin (KRT, KRT23, KRT19, KRT8, KRT7, and KRT5) and uroplakin (UPK, UPK1A, UPK1B, UPK2, and UPK3B) gene families were used to identify urothelial cells. Expression of VIM, SPARC, and DCN was referred to as interstitial (mesenchymal) cells. Co‐expression of TAGLN, DESM CNN1, ACTG2, and ACTA2 was used as markers of smooth muscle cells. Cells expressed PECAM1, VCAM1, and CDH5 were classed into the endothelial cell type. CDH19, GPM6B, S100B, and MPZ were used for marker genes of Schwann cells, while GPM6A, SLPI, MSLN, and RSPO1 were for neurons. Immune cells were divided into T cell (CD3D and CD3E), B cell (CD79A and MZB1), monocyte (CD14 and LYZ), and macrophage (CD74, HLA‐DRA, and C1QB).

### 3.3 Assumed biological activity scores across cell types

Similar to the default calculation function of cell‐cycle scores in the Seurat package, curated gene lists were employed to create activity scores of glucose, lactate metabolism, hypoxia, histone 3 (H3) acetylation, histone (H) methylation, and M6A. After scaled to 1–6 (6 for highest score) using different color bars by default method, scores were showed in the feature plot (Figure 1C‐D). In mouse bladder, glucose score was significantly high in most urothelial cells and part of smooth muscle cells, while lactate score had a similar but slighter trend. Hypoxia score was relatively low in urothelial cells (especially for urothelial cells 3), and the score of H3 acetylation was roughly equally distributed across cell types, while histone methylation and M6A scores were high in urothelial and endothelial cells. Though lacking urothelial cells, a similar pattern was seen in the human bladder; for example, H3 acetylation scores were evenly distributed among cells, and hypoxia score was higher in fibroblasts and immune cells. Glucose score was also elevated in immune cells and urothelial cells (a small population containing 17 cells) in the human bladder.

These scores were much more consistent inside the same tissue type (epithelial or interstitial tissues) than across different tissue types. Serial of H3 acetylations (H3K4ac, H3K9ac, H3K23ac, and H3K27ac) had already been recognized as major regulators of transcription activation.^20^ In our study, the score of H3 acetylation varied for individual cells inside a specific cell cluster but seemingly had a similar pattern across all cell types, which may indicate that transcriptional levels pattern at different tissues might be resembled. However, discrepancies of metabolic and hypoxia levels among tissues were easily observed, representing the fact that each kind of tissue had its unique function and morph. These phenomena also raised a question that evidence found by bulk RNA‐seq data at a prior era that could not locate physiological and pathological changes on a specific cell cluster may be worthwhile for re‐study again at the single‐cell level.

### 3.4 Differentially expressed genes and pseudotime trajectory of pan‐fibroblasts in mouse and human bladder

**FIGURE 2:**
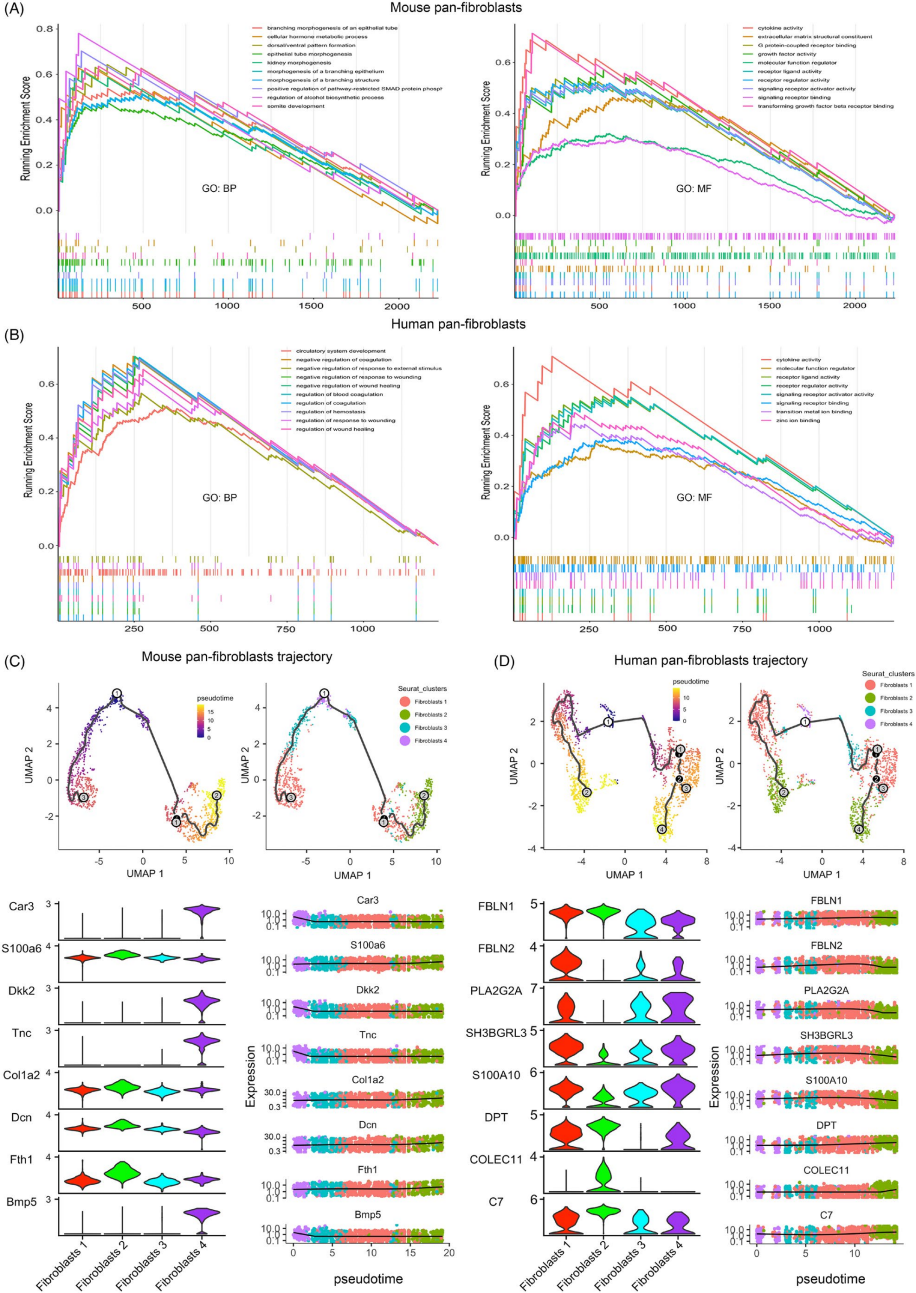
DEGs, GSEA, and trajectory analysis of pan‐fibroblasts in initial two datasets. (A) GSEA of DEGs from the mouse pan‐fibroblasts dataset. (B) GSEA of DEGs from the human pan‐fibroblasts dataset. (C) Trajectory plots, expression, and pseudotime patterns of top genes in mouse bladder. (D) Trajectory plots, expression, and pseudotime patterns of top genes in mouse. DEGs: differentially expressed genes. GSEA: gene set enrichment analysis. GO: gene ontology. Abbreviations: BP, biological process; MF, molecular function

We subset original datasets (Seurat clusters) to create pan‐fibroblasts datasets and then did differentially expressed genes (DEGs) analysis also by the “FindAllMarkers” function. Mouse pan‐fibroblasts (fibroblasts 1–4) subset contained 1471 cells, while human (fibroblasts 1–4) subset had 1961 cells. After the removal of duplicate cluster marker genes, there were about 2,200 and 1,400 DEGs in mouse and human, respectively, and these genes were descending ordered by log fold change (logFC) for the next step of GSEA. GSEA was done by “gseGO” function specifically for gene ontology (GO) analysis based on GO: molecular function (MF) and GO: biological process (BP) gene set collections in both mouse and human bladder. Pathways associated with the top positive normalized enrichment score (NES > 0) are shown in Figure 2A‐B. In mice, these DEGs were significantly enriched in several morphogenesis processes and also involved in cytokine and a series of receptor activity pathways. In human, DEGs were related to multiple biological functions of wounding, wounding healing, response to external stimulus, coagulation, and also a variety of receptor activity pathways.

Data of pan‐fibroblasts were import into Monocle 3 package from Seurat objects, and pseudotime trajectory analysis was constructed (Figure 2C‐D) following standard workflow with default parameters. In mouse bladder, fibroblast 4 seemed to be the beginning cells, fibroblasts 1 and 3 played intermediate roles, and fibroblasts 2 would be the end of the trajectory. We also extracted several top genes via a pseudotemporal pattern, among them, Car3, Dkk2, Tnc, and Bmp5 were highly expressed in the early stage, and S100a6, Col1a2, and Fth1 were in the later stage. We also depicted the distribution of these genes via four clusters. Car3, Dkk2, Tnc, and Bmp5 were almost exclusively expressed in fibroblasts 4 cluster. In the human bladder, fibroblasts 4 was at the beginning point, fibroblasts 1 and 3 were at the middle ways, while fibroblasts 2 were located at the end of development. Top pseudotemporal genes including FBLN2, PLA2G2A, SH3BGRL3, and S100A10 that highly expressed in early and/or middle stages and DPT, COLEC11, and C7 in the later stage of trajectory. Fibroblasts 2 exclusively expressed COLEC11 but lacking FBLN2 and PLA2G2A expressions, while fibroblasts 3 did not express DPT. Notably, the core pseudotemporal genes associated with fibroblasts trajectory seemed to be different across species (mouse and human). Furthermore, a previous article showed that Tnc and Bmp5 exhibited a cross‐organ similarity in Tnc + Cd34− fibroblasts in the colon and bladder of the mouse, while Dkk2 displayed a co‐expression pattern with Tnc in the mouse bladder.^14^ In addition, PLA2G2A, SH3BGRL3, and S100A10 have also been reported as top pseudotemporal genes in the human bladder from another study.^8^ Together, these data supported the repeatability and the robustness of our trajectory analysis and reflected potential organotypic or species differences.

### 3.5 Identification of common trajectory genes of urothelium and pan‐fibroblasts in bladder based on multiple non‐integrated datasets

Using the same workflow, we further processed another four mouse and five human bladder samples independently. After quality control, there were 2198 (GSM2889480), 504 (GSM3040905), 6134 (GSM3723360), and 6019 cells (GSM3723361) in mouse bladder and 205 (GSM3723357), 2556 (GSM3723358), 8407 (GSM3723359), 3760 (GSM3980126), and 5112 cells (GSM3980127) in human bladder to enter the clusters’ identification stage. The heterogeneity of dataset scales and cell proportions was obviously observed across different platforms and experimental proposals. UMAP plots and clusters’ identity of each dataset are displayed in Figure S1. GSM3723357 was removed for the next step of trajectory analysis due to insufficient overall cell amount (n = 205), and GSM3980126 was not subset for urothelial cells because of limited cell number (n < 30). After all, sub‐datasets of four mouse pan‐fibroblasts (n = 1,355, 258, 1,743, and 1,774), four mouse urothelial cells (n = 448, 197, 3,739, and 3,705), four human pan‐fibroblasts (n = 2,003, 949, 1,733, and 1,385), and three human urothelial cells (n = 359, 6,258, and 1,476) were ultimately investigated by trajectory analysis using monocle 3.

**FIGURE 3:**
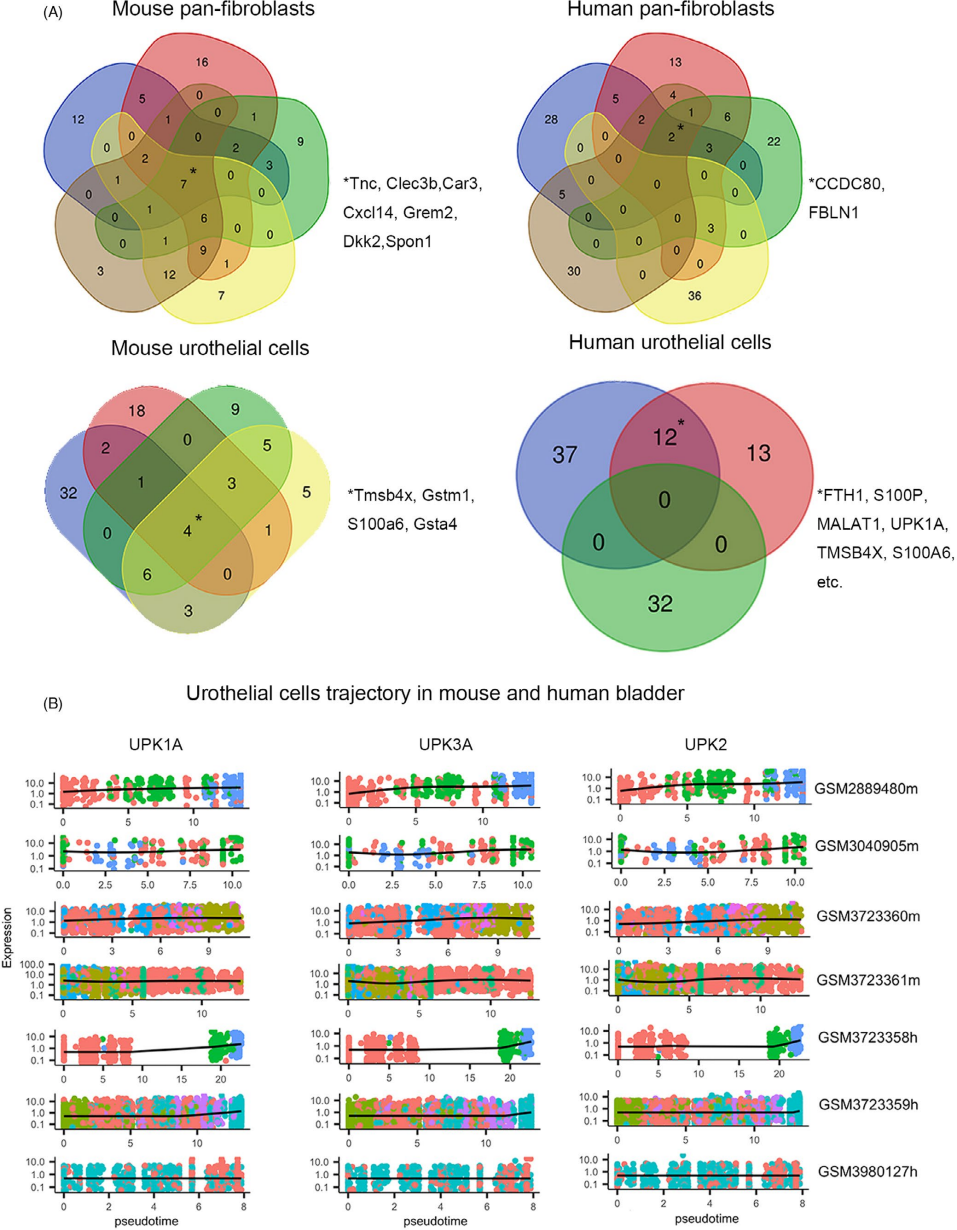
Venn plots of common top pseudotime genes. (A) Venn plots of mouse pan‐fibroblasts (n = 5), human pan‐fibroblasts (n = 5), mouse urothelial cells (n = 4), and human urothelial cells (n = 3) datasets. (B) Key pseudotime genes in both mouse and human urothelium tissues

Using Venn plots (Figure 3A), we identified several inter‐datasets common genes via pseudotime of pan‐fibroblasts or urothelial cells. Tnc, Clec3b, Car3, Cxcl14, Grem2, Dkk2, and Spon1 were found significant in all mouse pan‐fibroblasts datasets. CCDC80 and FBLN1 were found in four (out of five) human pan‐fibroblasts datasets. Similarly, Tmsb4x, Gstm1, S100a6, and Gsta4 were found significant in all mouse urothelial cells, and EIF1, TPT1, FTH1, UPK1A, TMSB4X, S100A6, etc. were simultaneously found in two (out of three) human urothelial cell datasets. Then, we tried to explore the heterogeneity and homogeneity of chosen genes among datasets and species. First, we noticed that some genes (such as Car3 and Dkk2) significantly showing a pseudotime pattern in mouse pan‐fibroblasts even could not be found in the scRNA‐seq expression matrix of their human counterparts. Furthermore, Cd34 and Tnc were reported as markers of differentially located fibroblasts in mouse and showed a strong pseudotime pattern in mouse pan‐fibroblasts datasets but not in human, while FBLN1 was a top gene in human pan‐fibroblasts datasets but not in mouse (Figure S2). Second, only a few candidates (eg, S100A6 and TMSB4X for urothelial cells) were considered as shared pseudotime genes in both human and mouse (Figure S3A‐B). Unfortunately, the pseudotime trends of S100A6 and TMSB4X seemed to be slight across all datasets, even though they still hit the statistical threshold. Thirdly, even if a certain gene was found significant in all datasets,its pattern would easily differ from the different data sources. CXCL14 and DCN seemed to have an inconsistent pattern in pan‐fibroblasts among datasets and between species (Figure 3C‐D). However, we found out that UPK1A (Upk1a), UPK3A (Upk3a), and UPK2 (Upk2) were consistently highly expressed in the end stage of urothelial cells (Figure 3B) in both mouse and human datasets. High expression of these genes was referred to as markers of umbrella cells (most superficial cells of urothelium) in the bladder. Such results reminded us that trajectory analysis could at least partially capture the key genes via pseudotime characteristics, but the heterogeneity between datasets and species might need further advanced algorithms to solve.

### 3.6 Integrated datasets of mouse and human bladder

**FIGURE 4:**
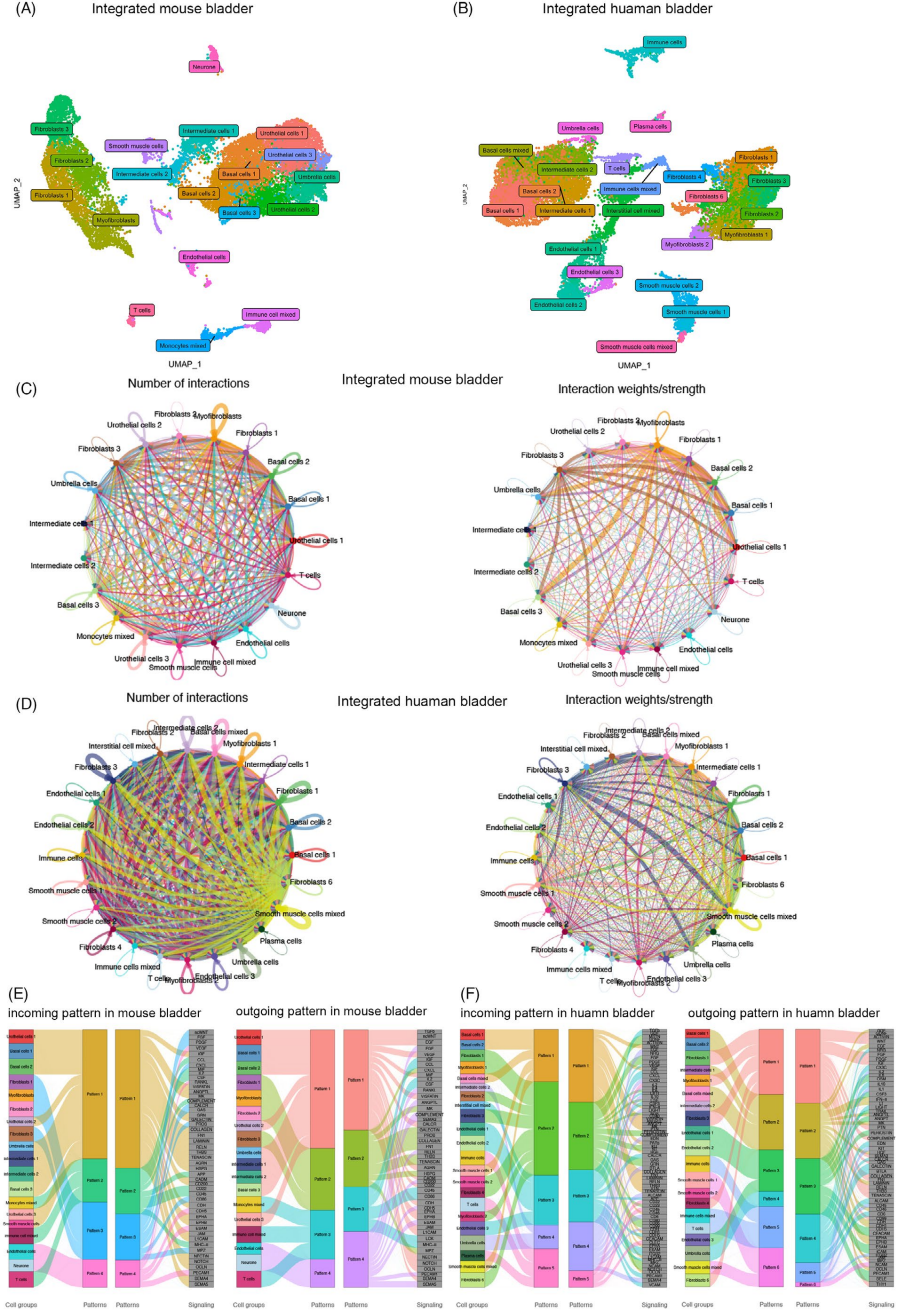
Integrated mouse and human bladder datasets. (A) UMAP of integrated mouse bladder. (B) UMAP of integrated human bladder. (C) Cell‐cell interactions in mouse. (D) Cell‐cell interactions in human. (E) Classification of cell clusters based on incoming or outgoing signaling patterns in mouse. (F) Classification of cell clusters based on incoming or outgoing signaling patterns in mouse

Besides the above independent analysis on each dataset respectively, we tried to integrate these datasets (not including GSM4201633 and GSM4850577) created from different platforms and experimental protocols. After integration, 16,688 and 22,080 cells of mouse and human normal bladder, respectively, were finally analyzed. There are 19 and 24 cell clusters identified in mouse and human combined datasets, respectively, and displayed using UMAP nonlinear reduction with representative marker genes in Figure 4A‐B and Figure S4A‐B. In general, these clusters were roughly accordant with the analysis of the above independent dataset and thus mainly consisted of urothelial (eg, umbrella, intermediate, and basal cells), interstitial (eg, fibroblasts and myofibroblasts), smooth muscle, immune cells (eg, T and plasma cells), and others (neurone and endothelial cells) in the normal bladder. The majority of captured cells were urothelial cells in mouse (57.2%, 9,553/16,688) and human (42.6%, 9,396/22,080) integrated datasets.

### 3.7 Cell‐cell communication in integrated datasets

Then, we performed cell‐cell interaction analysis by the CellChat R package to further explore the dynamic cross talk in the bladder. There were 1330 and 1449 interactive pairs verified by cell‐cell communication analysis in mouse and human groups, respectively. The total number and weight of interactions between cell clusters are shown in Figure 4C‐D. Within it, 55 (in mouse) and 95 (in human) pathways were involved. According to incoming communication patterns of target cells, cell groups were clustered into four classifications in mouse datasets and five classifications in human datasets, while for outgoing patterns, four classifications in mouse datasets and six classifications in human datasets were found (shown in Figure 4E‐F). These patterns were based on a hierarchical clustering of the consensus matrix of incoming or outgoing signaling pathways. When several cell clusters together went into one pattern, we can assume that these cell clusters shared many same pathways. So, these classifications were strongly associated with specific tissue types (eg, all immune cells went into pattern 3 and all fibroblasts went into pattern 2 in outgoing communication analysis of mouse bladder), and to some extent, this, in turn, proved that the previous cell clustering of integrated datasets based on Seurat was proper. The details of significant contributing signals of incoming and outgoing patterns in all cell clusters are displayed in Figure S5A‐B.

### 3.8 Interactions between urothelial basal cells and fibroblasts in mouse bladder

**FIGURE 5:**
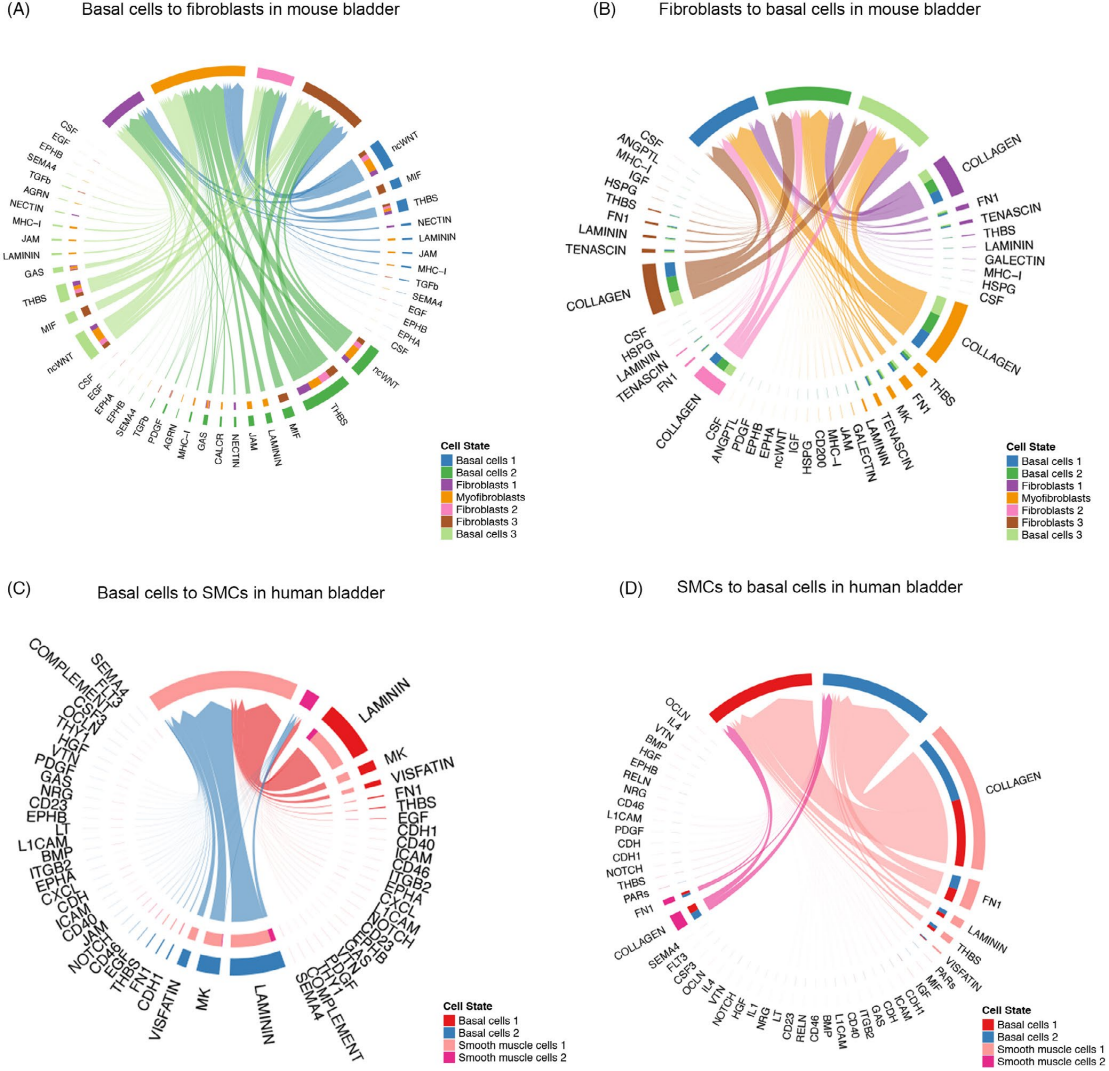
Cell‐cell communications between certain cell types. (A) Signaling from basal cells to fibroblasts in mouse. (B) Signaling from fibroblasts to basal cells in mouse. (C) Signaling from basal cells to SMCs in human. (D) Signaling from SMCs to basal cells in human. SMCs: smooth muscle cells

**FIGURE 6:**
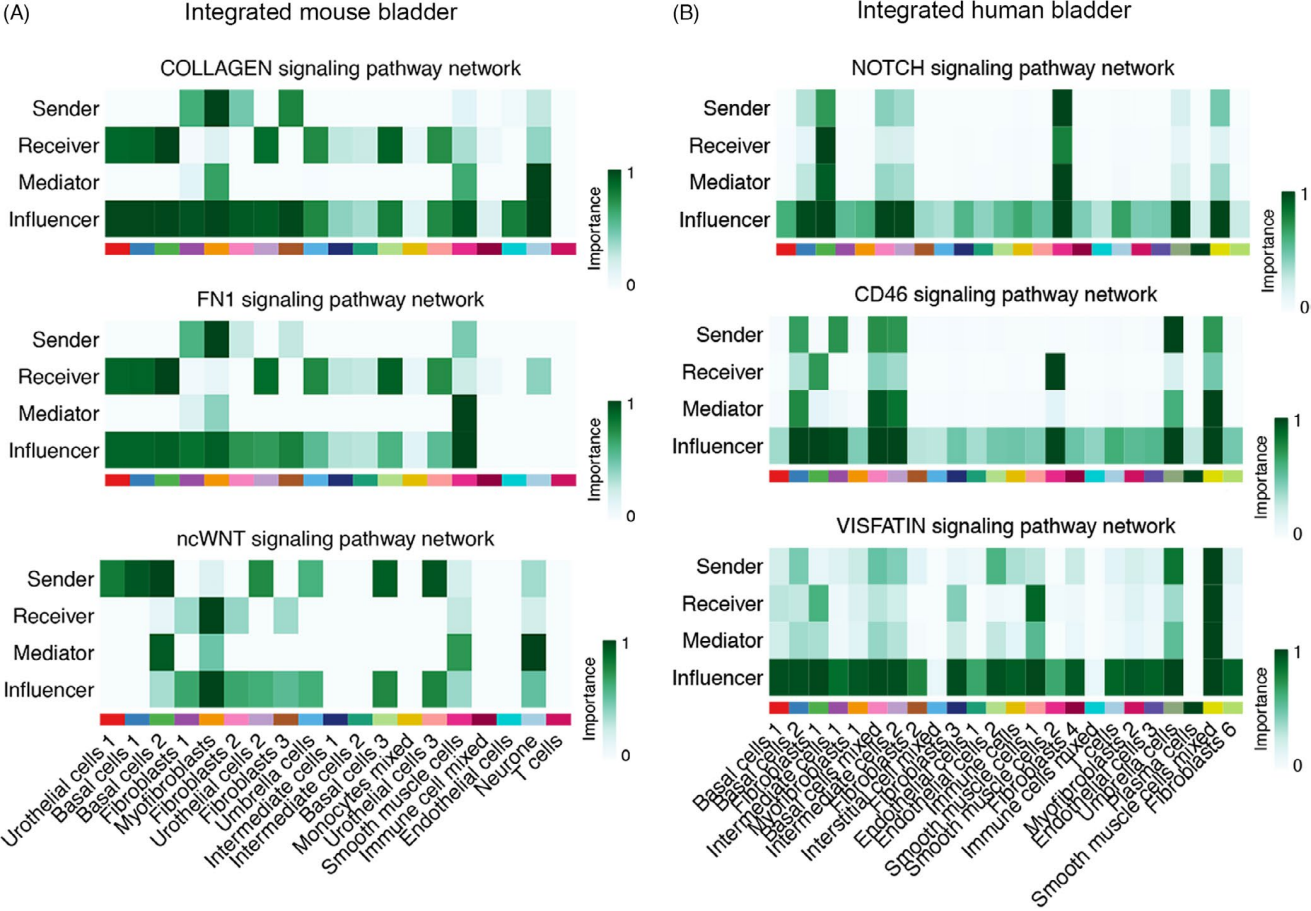
Different roles of each cell cluster in certain pathways. (A) Pathways in the integrated mouse dataset. (B) Pathways in the integrated human dataset. ncWNT: non‐canonical WNT. Abbreviation: FN1, fibronectin 1

We further explored the potential communication in depth between urothelial basal cells (basal cells 1–3) and fibroblasts (fibroblasts 1–3 and myofibroblasts) in mouse bladder, since these cells were most likely physically close based on the anatomical structure of the bladder. First, we identified the common significant interactive pathways in these cells, by setting fibroblasts as source cells and basal cells as targeted cells, and vice versa (Figure 5). The outgoing signals from basal cells to fibroblasts included non‐canonical WNT (ncWNT), macrophage migration inhibitory factor (MIF), Nectin pathways, and several extracellular matrix (ECM) receptor pathways such as thrombospondin (THBS) and laminin. The incoming signals from fibroblasts to basal cells included collagen, fibronectin 1 (FN1), tenascin, midkine (MK), and galectin pathways. Then, we also confirmed the roles of other cell clusters in some pathways we were interested in (Figure 6A and Figure S6A‐B). In the collagen signaling network, fibroblasts, especially, myofibroblasts were the senders, all urothelial cells were receivers, and this pathway was influenced by almost all cell clusters except for immune cells. In the FN1 pathway, myofibroblasts were the strongest sender, urothelial cells (especially basal cells) were the receivers, and it was only heavily mediated by smooth muscle cells. In the ncWNT signaling network, urothelial cells were the strong senders; myofibroblasts were the major receiver.

### 3.9 Interactions between urothelial basal cells and smooth muscle cells in human bladder

In addition, we discovered the estimated interactions between the bottom of the urothelium (basal cells 1–2) and the certain type of smooth muscle cells (detrusor) in the human bladder. Using similar processes, we explored the interactions when smooth muscle cells were targets and basal cells were sources, and vice versa (Figure 5). The bi‐directional interactions between basal cells and smooth muscle cells included THBS, MK, FN1, visfatin, and laminin pathways. The collagen pathway was the most abundant signaling from smooth muscle cells to basal cells. We then selected a few pathways that significantly contribute to smooth muscle cells and explores the different roles of all cell clusters (Figure 6B and Figure S6C‐D). Interestingly, in the Notch pathway, the cluster of smooth muscle cells 2 was the key sender while smooth muscle cells 1 did not serve the same function, and fibroblasts 1 was the main receiver while other fibroblasts did not act the same role. Smooth muscle cells 2 also played a significant role as a receiver in the CD46 pathway network, while smooth muscle cells 1 did not. However, smooth muscle cells 1 were the receiver for the visfatin signaling network, while smooth muscle cells 2 was not.

### 3.10 Interactive networks of integrin superfamily in the bladder and its adhesion to cells and extracellular matrix

**FIGURE 7:**
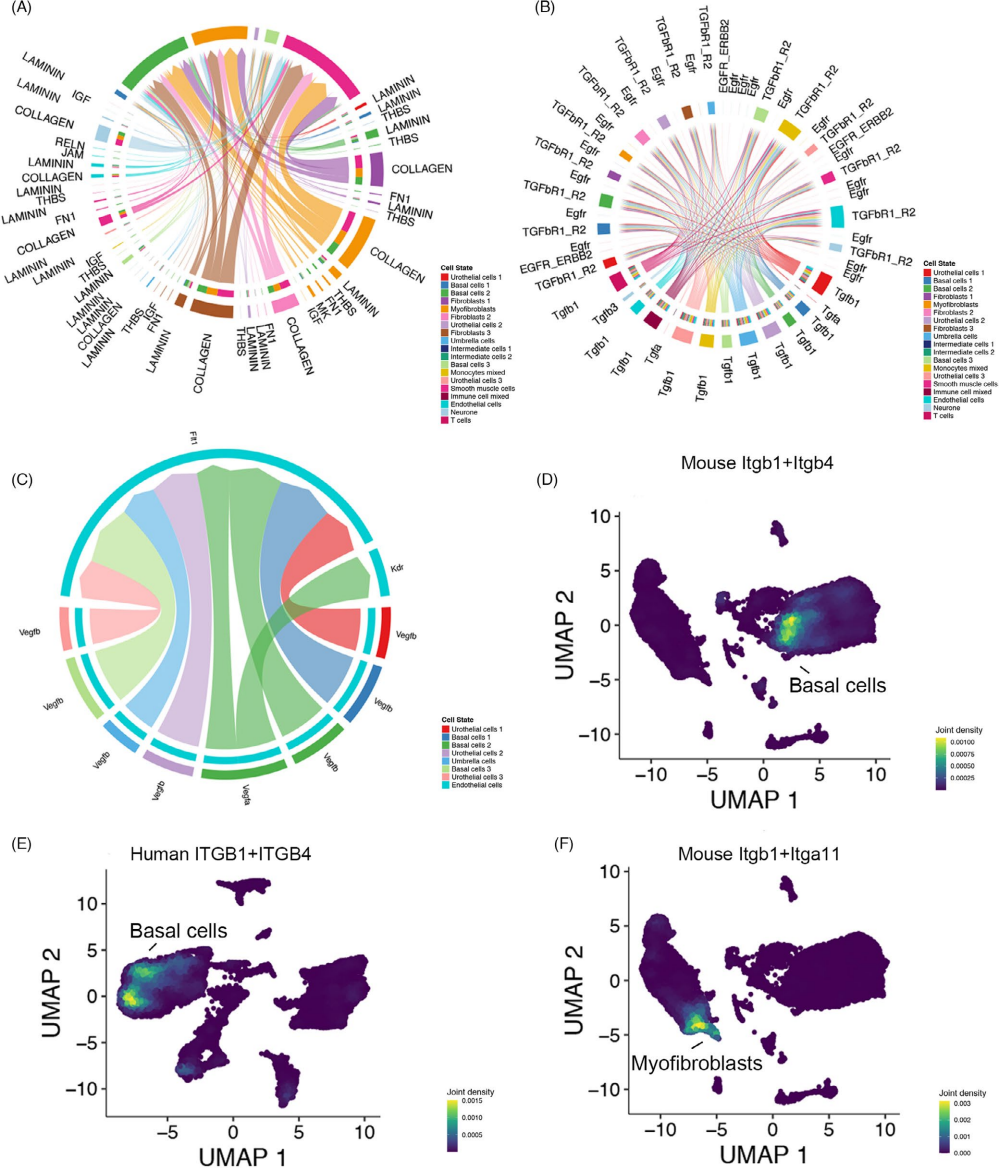
Distributions of certain interactive pathways in bladder and co‐expression of integrin superfamily. (A) Communication networks of integrin superfamily in mouse bladder. (B) Communication networks of the TGF pathway in mouse bladder. (C) Communication networks of the VEGF pathway in mouse bladder. (D) Co‐expression of Itgb1 and Itbg4 in mouse bladder basal cells. (E) Co‐expression of ITGB1 and ITGB4 in human bladder basal cells. (F) Co‐expression of Itgb1 and Itga11 in mouse bladder myofibroblasts. TGF: transforming growth factor. Abbreviation: VEGF, vascular endothelial growth factors

Integrin superfamily contains 18 αsubunits (ie, ITGA1, ITGAV, ITGA6, ITGAX) and 8 βsubunits (ie, ITGB1 and ITGB2) generating 24 distinct integrin heterodimers.^21^ These transmembrane receptors are mainly responsible for connections between “outside” ECM structures and “inside” cell cytoskeleton systems and are also able to co‐operate with cell‐cell junction signaling.^22^ Among all enriched ligand‐receptor pairs found by our interactive communication analysis, 24.7% (66/268) receptors in mouse and 23.9% (102/426) receptors in human were directly associated with the integrin superfamily, suggesting its critical role in cell communication networks. We extracted all integrin‐associated receptors from different significant enriched pathways and showed their networks in the mouse bladder (Figure 7A). Urothelial cells (basal cells) and myofibroblasts were the main receivers of these signaling, while most other cell clusters send related ligands. In addition, we were using the other two pathways as a comparative reference (Figure 7B‐C). Vascular endothelial growth factor (VEGF) signaling was exclusively from urothelial cells to the endothelial cells, while transforming growth factor (TGF) signaling was equally distributed to almost all cell clusters in mouse bladder.

Then, we browsed the expression of these subunits in mouse (all 26 subunits) and human (25 subunits, ITGAD not available) bladder (Figure S7A‐B). In both mouse and human, ITGB2 was primarily expressed in immune cells, ITGB1 was the most broadly expressed subunit gene, and ITGA1 was only expressed in smooth muscle and endothelial (potentially include pericytes) cells. Co‐expression of ITGB1 and ITGB4 was located in basal cells in both mouse and human, implying this layer was tightly connected to the basement membrane (Figure 7D‐E). Co‐expression of Itga11 and Itgb1 has precisely occurred in myofibroblasts of mouse bladder (Figure 7F). Meanwhile, some differences between species have been observed; myofibroblasts in the human bladder not only highly expressed ITGA11 but also relatively highly expressed ITGA8 when myofibroblasts in mouse bladder only highly expressed Itga11. The overall expression of ITGA2 was significantly lower in the mouse bladder. Itgb7 was highly expressed in immune cells of mouse bladder, while ITGB7 was barely expressed across all human bladder cell clusters. Taken together, these findings were consistent with the previous reports,^21^, ^22^, ^23^ except for a few potential organs or species specificities.

## 4 DISCUSSION

In recent years, whether it is the construction of large‐scale cellular atlases or the analysis of single‐cell data from a small number of samples, we are trying to understand cell fate, development, and communication at an unprecedented depth, so as to ultimately grasp and interpret the underlying mechanisms of phenomena that can be observed by us even at the naked eye level. In our study, we eventually collected and analyzed more than 23,000 mouse cells and 29,000 human cells from 11 bladder samples (six different scRNA‐seq datasets). Notably, sample isolation and processing, along with the different scRNA‐seq platforms, would significantly affect the results. Rapid and efficient sample processing and non‐over‐digested single‐cell suspensions might be a prerequisite to maintain favorable cell status (relatively low mitochondrial or ribosomal contamination) and facilitate subsequent data analysis. Followed by standard Seurat package workflow, we suggested a resolution between 0.3 and1 to find distinct cell clusters for bladder cells ranging from 3,000 to 20,000 (Figure S8). Normally, a higher resolution would only create more sub‐clusters of urothelial cells or fibroblasts, and whether these sub‐groups of cells exist unique features was still unknown since there was no widely accepted optimal resolution for distinguishing different cell groups. Interestingly, it appeared that more immune cells can be detected in human samples, and we suspected that this could be related to the germ‐free environment in which the mice were raised and the short laboratory animal lifespan.

The canonical marker genes of most cell clusters in the bladder were well established. However, we found that sometimes it was difficult to distinguish myofibroblasts from smooth muscle cells when a group of cells highly expressed ACTA2 and barely expressed fibroblast markers. Also, it would be challenging to decipher immune cells in the bladder when the cell amount was limited and marker genes were mixed. As a stratified epithelium, urothelium is typically comprised of three cell layers including umbrella cells (most superficial), intermediate cells (middle layer with one‐to‐several layers thick), and basal cells. For instance, KRT5 and KRT17 were previously reported as markers of basal cells.^8^ But, the expression of KRT17 was not detected in our initial dataset analysis, and it might be caused by a common drop‐out effect in scRNA‐seq experiments. UPK2 and KRT20 were regarded as umbrella cell markers^8^ while in some datasets both of them could hardly express across all urothelial cells, and it can be tough to determine whether a specific cell cluster belongs to umbrella cell with underestimated UPK2 and KRT20 expression or the whole sample was missing the superficial layer of bladder probably by inappropriate experimental protocols. KRT13, KRT18, KRT19, and relatively low expression of UPK2, UPK1A, UPK1B, and UPK3B were viewed as markers of intermediate cells, and the previous research exhibited a potential transitional status between intermediate and umbrella cells.^7^, ^8^

Metabolic patterns were one of the customized features for a certain cell cluster. We found that urothelial cells seem to have a higher glucose metabolic activity score, implying that they were in a constant state of renewal and, therefore, have a high energy demand. The hypoxia score was higher in fibroblasts, smooth muscle cells, endothelial cells, and some immune cells. These cells are traditionally thought to be mainly responsible for hypoxia, and targets are driven by hypoxia conditions in a tissue.^24^, ^25^ Although the score built on the gene‐list expression method only partially represented the real activity status, it still reminded us that heterogeneities across cell types that cannot be ignored and thus previous studies based on bulk RNA‐seq might have severe confounding factors (ie, mixed cell types) and then mask the truth.

Fibroblasts are known to be mesenchymal origin cells and comprise the majority of interstitial cells in the bladder^26^ with undisputed important biological functions, especially for tissue fibrosis, wound contraction, and the formation of extracellular matrix.^14^ DEGs were intensely associated with morphogenesis pathways, which was expected, as fibroblasts somehow could shape the water content and tensile properties in tissues.^14^ Tightly connected to the ECM, these enriched pathways were also correlated with multiple receptor signaling. As we can see, these DEGs‐enriched pathways were nearly identical between mouse and human, representing a great similarity in fibroblast function between species.

In accordance with the previous findings, we validated that PLA2G2A, S100A10, and SH3BGRL3 were lowly expressed at the end stage of trajectory via a strong pseudotime pattern in the human bladder. PLA2G2A is a prominent marker of fibroblasts in the bladder, and its expression would be substantially reduced in bladder tissue from patients with prune belly syndrome.^27^ Also, evidence from human and rat lung tissue microarray data indicated that PLA2G2A was overexpressed in patients with idiopathic pulmonary fibrosis, and in our speculation, which was most likely due to an increase in the abundance of fibroblasts.^28^ ECM genes (eg, FBLN1 and FBLN2) also exhibited a pseudotime pattern in the human pan‐fibroblasts dataset, fibroblasts derived from patients with synpolydactyly (hand malformations) showed alterations in the level of FBLN1 splice variants,^29^ and ablation of Fbln2 in mice cardiac fibroblasts protected against progressive ventricular dysfunction, reducing the mortality after myocardial infarction.^30^ In mouse bladder, Car3, Dkk2, Tnc, and Bmp5 were among top trajectory genes with pseudotime expression features, overexpression of DKK2 would reduce the activation of human cardiac fibroblasts,^31^ Tnc was involved in modulating ECM integrity and preventing skin aging,^32^ and Bmp5 was an antifibrotic factor that related to fibroblast‐myofibroblast transdifferentiation in rat kidney interstitial fibroblasts.^33^

Cxcl14 and Grem2 consistently showed a pseudotime trait in all five mouse pan‐fibroblasts; previous studies displayed that the Cxcl14 axis in fibroblasts can interact with multiple cancer cells and acts as a multi‐modal stimulator with tumor‐supporting properties,^34^, ^35^, ^36^ and the activation of Grem2 in fibroblasts would promote pulmonary fibrosis.^37^ For the development of urothelial cells, TMSB4X was a top gene in all datasets regardless of the mouse or human tissue sources, depletion of TMSB4X would cause abnormal stability of adherence junction in epidermal cells,^38^ and a developmental trajectory using single‐cell proteomics revealed TMSB4X significantly decreased during hair‐cell differentiation.^39^ In addition, increasing expression of UPK2, UPK1A, and UPK3A (Figure 3B) has been seen through all datasets in both mouse and human bladder. Knockout of these genes in mice would cause several abnormalities, such as poorly differentiated umbrella cells and vesicoureteral reflux with hydronephrosis.^40^ In mouse embryonic day 11–12, progenitor cells of urothelium were formed with the expression of SHH, FOXA2, TP63, and uroplakins (most be UPK3A) but without KRT5.^40^ However, in adult mouse urothelium, UPK3A−, KRT5+, and KRT14+ basal cells were reported as stem cells with the ability to give rise to all urothelial cells and UPK3A+ intermediate cells can give rise to umbrella cells in some cases.^40^ Intriguingly, trajectory analysis of urothelial cells would often be disrupted by a large number of ribosomal genes which also developed pseudotime properties in some datasets. This observation was also seen in a previous study, about 90% of top pseudotime genes were located in ribosomes,^9^ yet the remaining genes (Gstm1, Tmsb4x, S100a6, and Malat1) were still aligned with our study. This phenomenon might imply that the urothelial cells may have been more heavily damaged during the sample preparation because of their exposure as the outermost layer or their intolerance and sensitivity to cell digestive agents (multiple enzymes).

To give a better perspective of the entire bladder, we have integrated the above datasets (two initial independent datasets not included) using the Seurat R package (CCA method) with slightly more looser quality control parameters compared with earlier independent analysis. In general, the results displayed that the integration appropriately addressed and merged the original cell subpopulations, underlying the major cell types in the mouse and human bladder (Figure 4A‐B). To our knowledge, this is the first study to integrate bladder scRNA‐seq data from different platforms, focusing on this specific organ, and thus produced the largest data of normal bladder at single‐cell levels. Then, cell‐cell communication analysis was conducted using a recently published R package CellChat.^19^ Notably, these dynamic interactive networks were broadly dispersed across cell types (Figure 4C‐D).

To further investigate intercellular communication between specific cell types, we have chosen basal cells and fibroblast in the integrated mouse dataset along with basal cells and smooth muscle cells in the integrated human dataset as examples (Figure 5A‐D). Bladder fibroblasts could promote re‐epithelization after urothelial injury through enhancement for cell proliferation, attachment to the basal lamina, and development of well‐organized cell junction between multilayered urothelial cells.^41^ Also, the existence of laminin, collagen, and elastin in the bladder submucosa matrix was maintained as valuable bioactive factors even after the decellularization and extraction processes.^42^ For these interstitial cells (eg, fibroblasts), their close proximity to the urothelium and smooth muscle cell (detrusor) seemed to suggest their modulating or bridging role in the bladder wall.^40^ Communication between human bladder smooth muscle cells and suburothelial myofibroblasts was directly associated with overactive bladder syndrome and could be profoundly affected by different cytokines.^43^ At last, we took integrin superfamily, TGF, and VEGF pathways as cases for illustrating the different roles and distribution patterns of signaling in mouse bladder (Figure 7). It is obvious and intuitive that the different patterns correspond to varying functions and localization of signaling pathways.

In summary, we collected multiple datasets to comprehensively dissect the bladder at a single‐cell level. To date, this is the first and largest integration study of the normal bladder using single‐cell transcriptome data. DEGs and pseudotime analysis of pan‐fibroblasts revealed similarity in function and potential distinct development trajectory between mouse and human bladders. Whether these heterogeneities are caused by any technical factors during scRNA‐seq needs further investigation. TMSB4X and S100A6 show a pseudotemporal signature in the multiple mouse or human urothelial cell datasets, and the specific roles they play need to be further examined. Tons of interactive communications could be recognized in our large‐scale integrated bladder datasets, and future studies could proceed to explore whether these paired signals are significantly altered under pathological conditions. Also, we provide information on which signaling pathways are enriched in particular cell clusters (eg, urothelial basal cells, fibroblasts, and smooth muscle cells) of the bladder and what roles (eg, sender, receiver, and mediator) different cells play in the pathways. The exact mechanisms of how these signaling pathways are synergistically regulated by a variety of distinct cells and function stably are worth further exploration.

## CONFLICT OF INTEREST

The authors declare no competing interests.

## AUTHOR CONTRIBUTIONS

Bowen Shi, Jie Ding, and Jun Qi designed the study; Yanyuan Wu, Haojie Chen, and Jie Ding collected the data; Bowen Shi performed the bioinformatic analysis. Bowen Shi and Jun Qi drafted the manuscript. Yanyuan Wu, Haojie Chen, and Jun Qi prepared the figures. All authors read and approved the final version of the manuscript.

## ETHICS APPROVAL AND CONSENT TO PARTICIPATE

Not applicable to this study.

## CONSENT FOR PUBLICATION

All authors read and approved the final version of the manuscript for publication.

## Supporting information

Fig S1

Fig S2

Fig S3

Fig S4

Fig S5

Fig S6

Fig S7

Fig S8

## Data Availability

The datasets used in the present study are available on the Gene Expression Omnibus (GEO, https://www.ncbi.nlm.nih.gov/geo/). The analysis code can be found in the GitHub repository (https://github.com/BowenShi‐scRNA/bladder‐scRNA‐seq).
