# Supplementary figures and images for: Understanding of mouse and human bladder at single‐cell resolution: integrated analysis of trajectory and cell‐cell interactive networks based on multiple scRNA‐seq datasets
Source: Cell Prolif. 2021 Dec 23;55(1):e13170. doi: 10.1111/cpr.13170 (PMC8780900; doi:10.1111/cpr.13170)

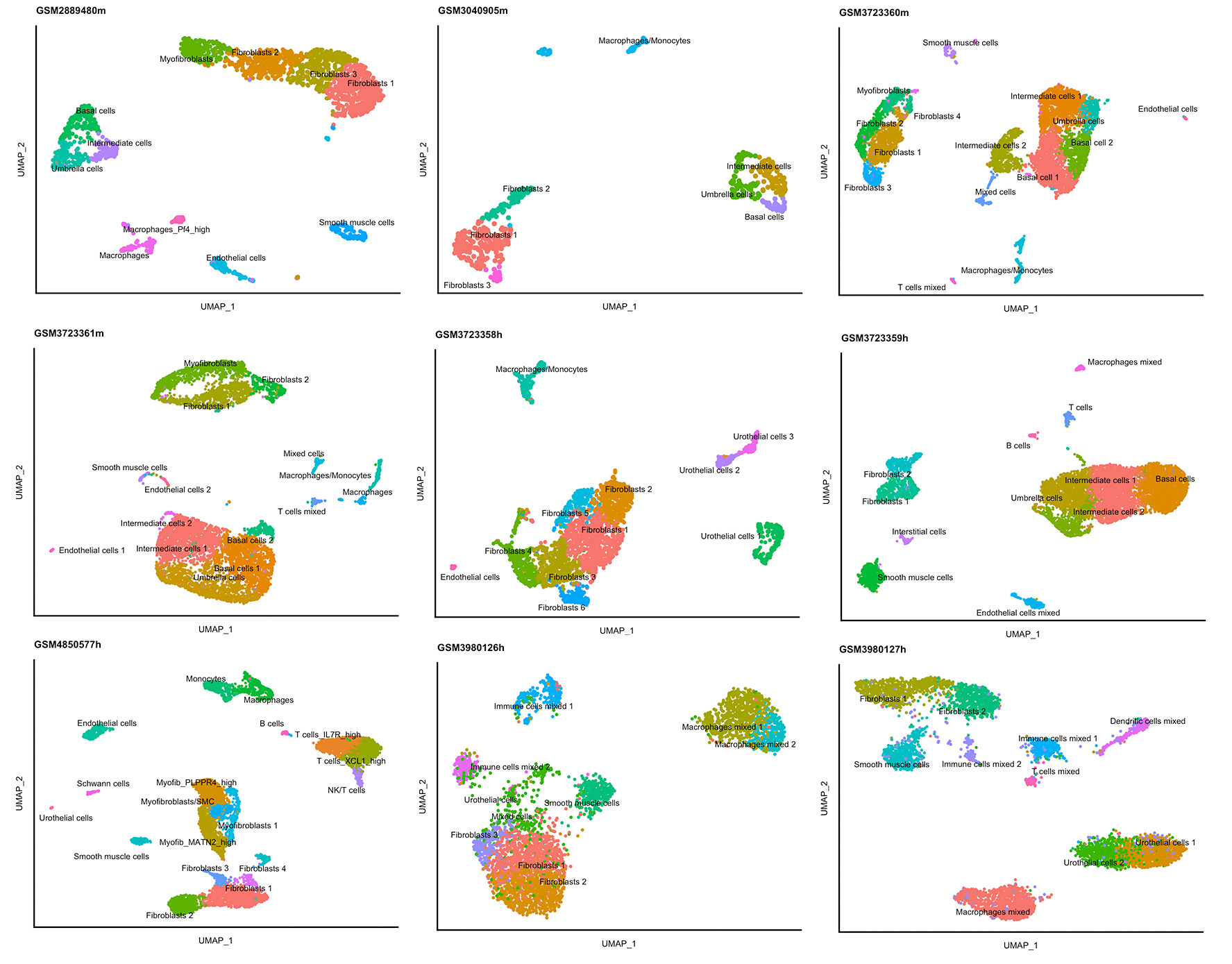

Supplement: Supplementary file 1 — Fig S1 [file CPR-55-e13170-s006.jpg]

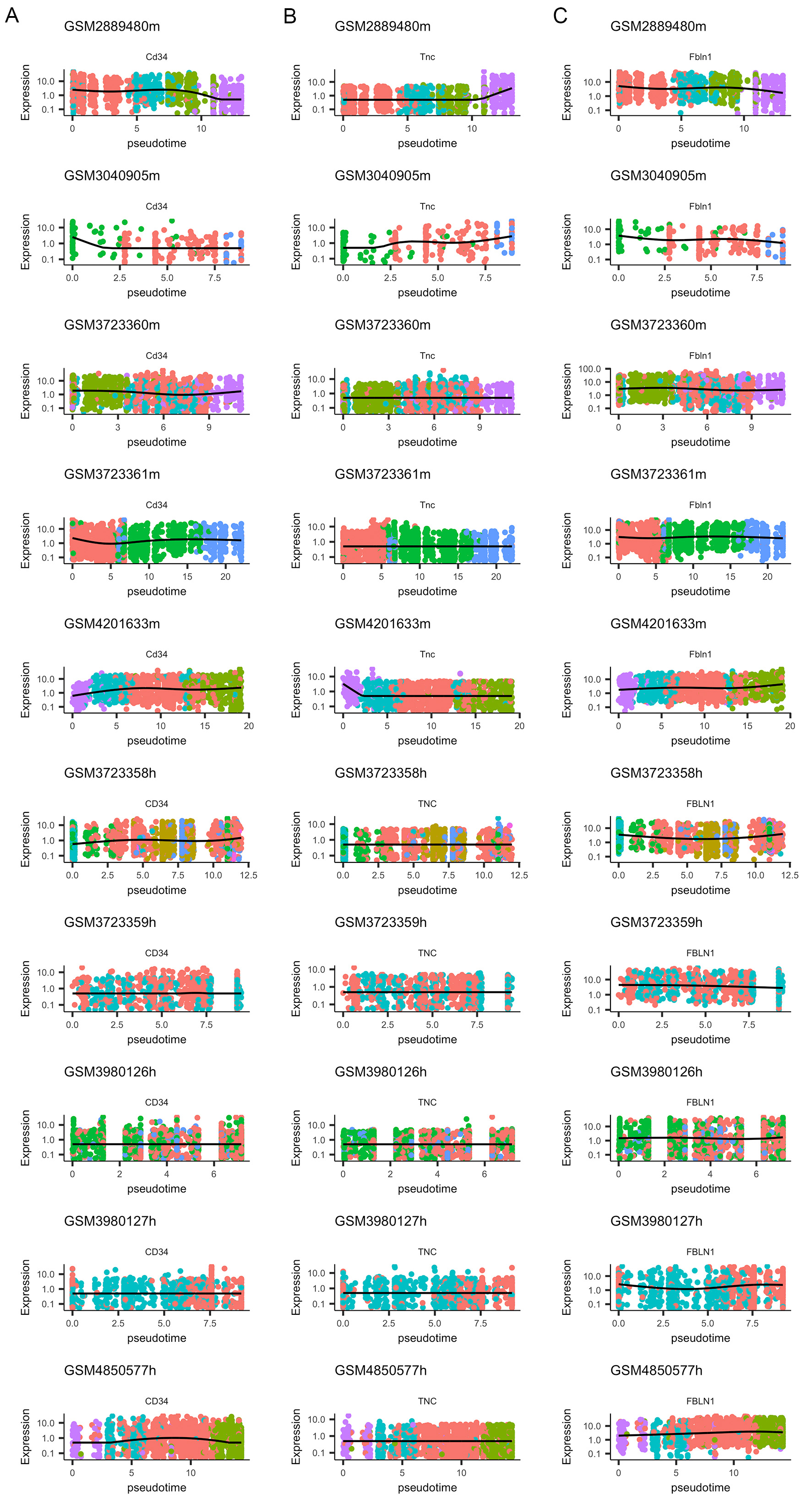

Supplement: Supplementary file 2 — Fig S2 [file CPR-55-e13170-s007.jpg]

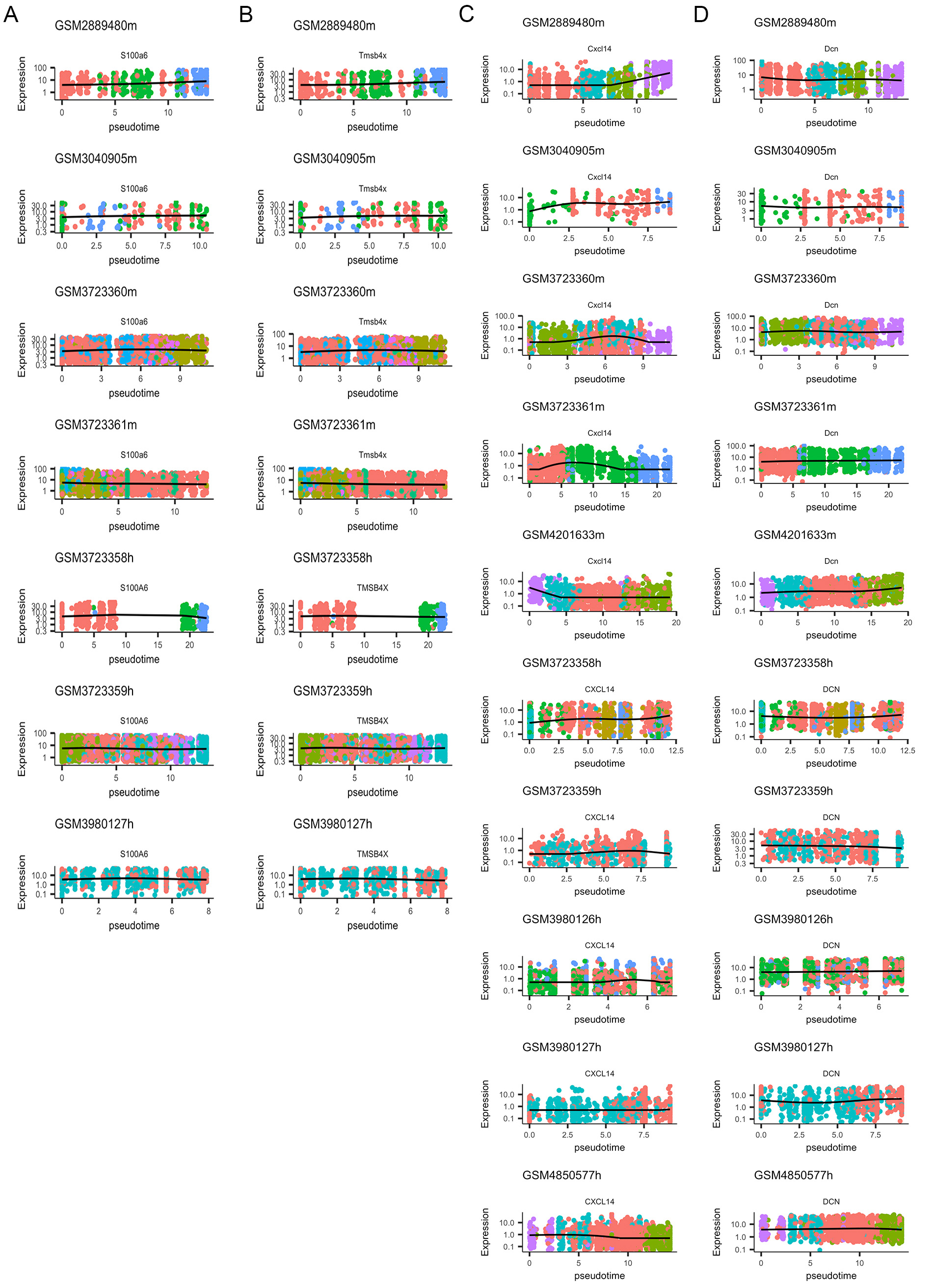

Supplement: Supplementary file 3 — Fig S3 [file CPR-55-e13170-s004.jpg]

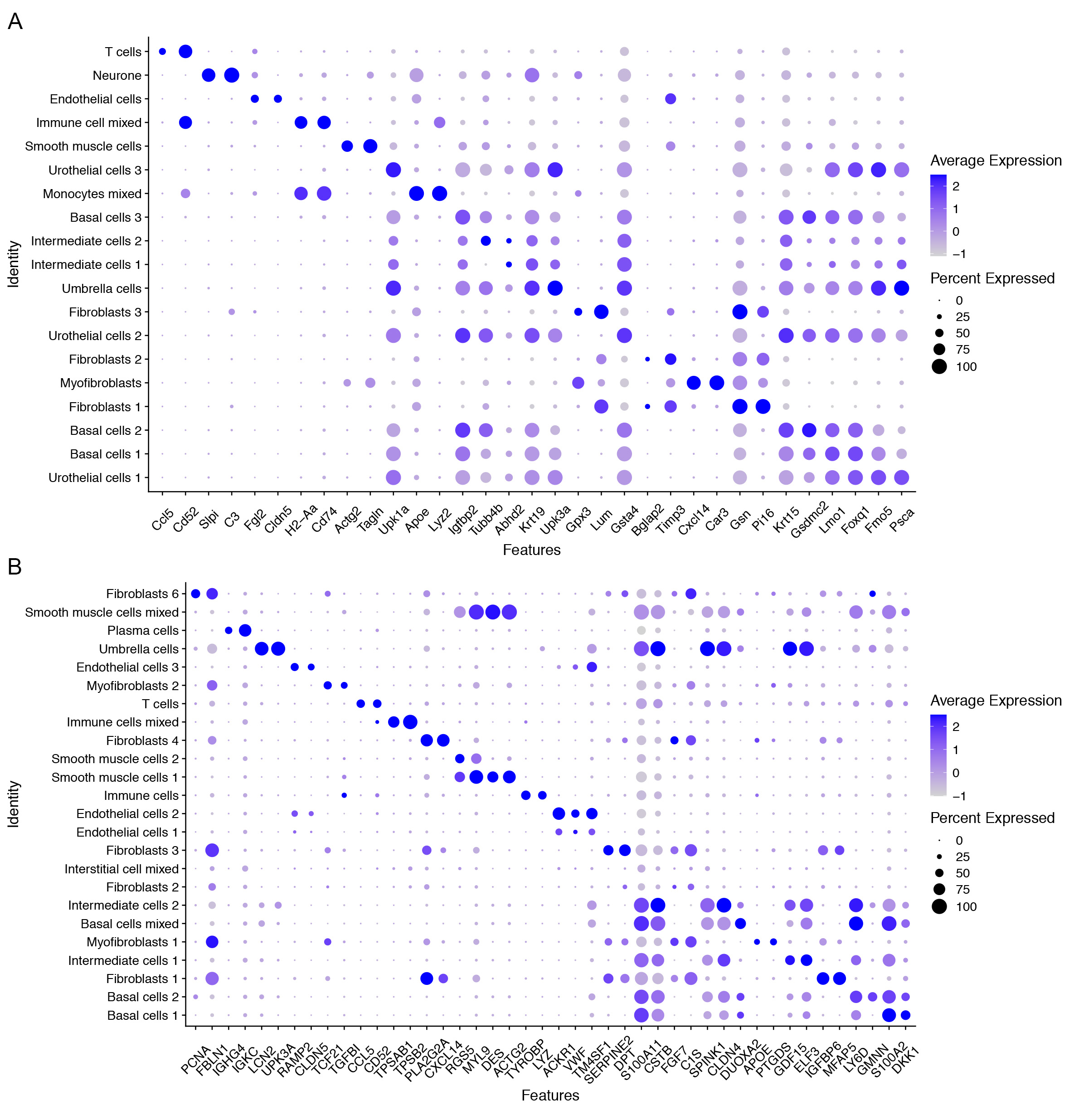

Supplement: Supplementary file 4 — Fig S4 [file CPR-55-e13170-s002.jpg]

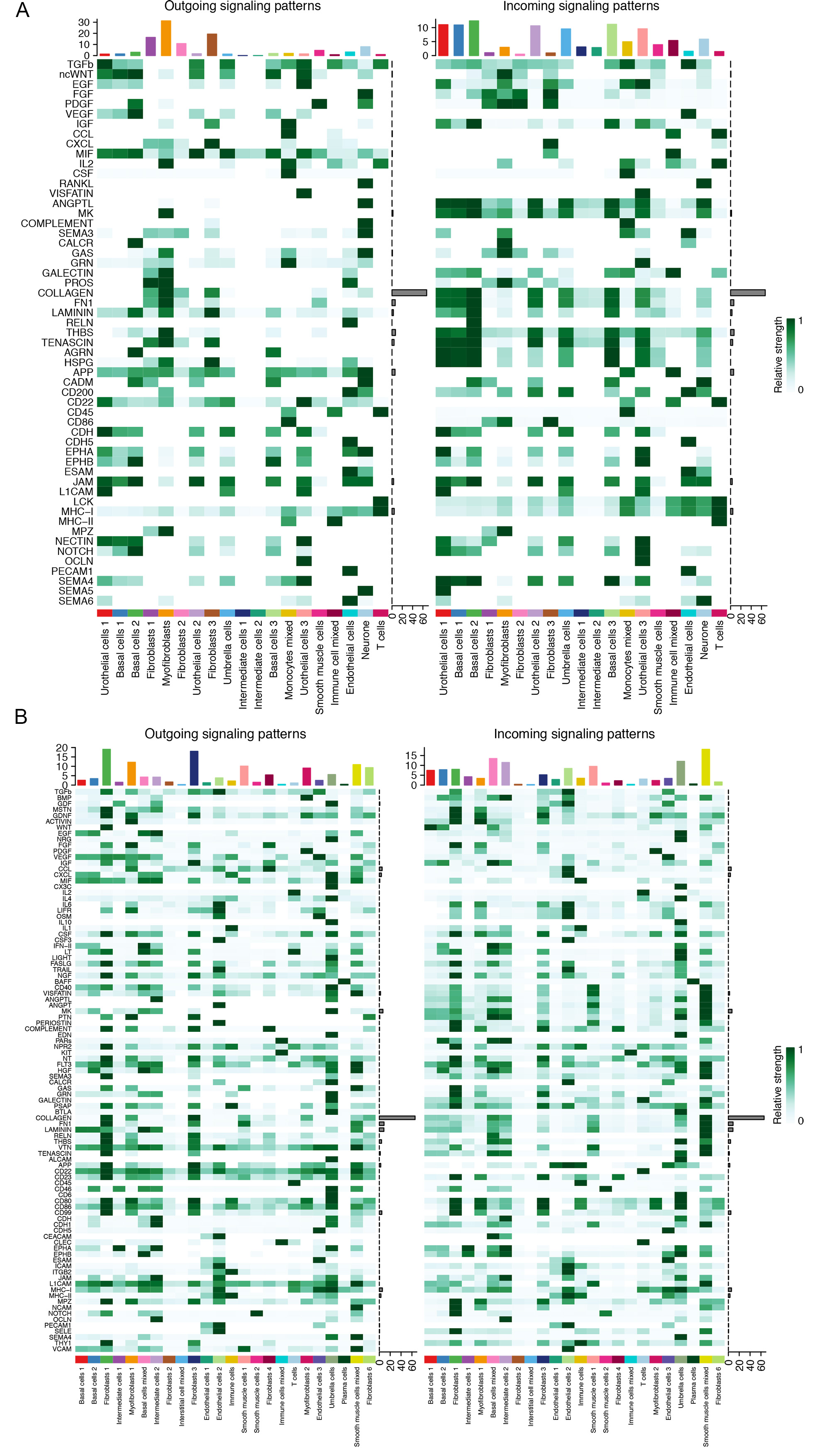

Supplement: Supplementary file 5 — Fig S5 [file CPR-55-e13170-s001.jpg]

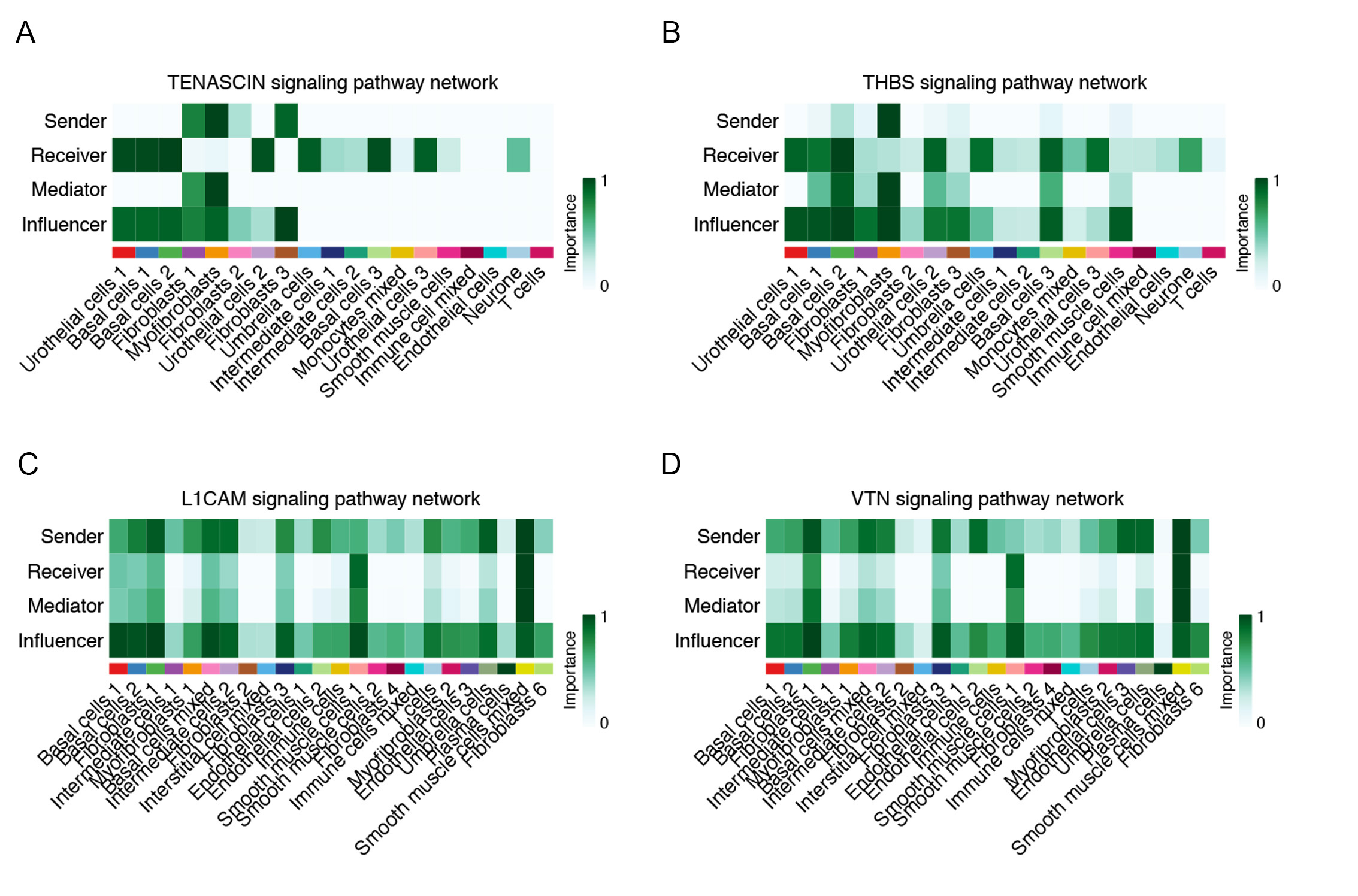

Supplement: Supplementary file 6 — Fig S6 [file CPR-55-e13170-s003.jpg]

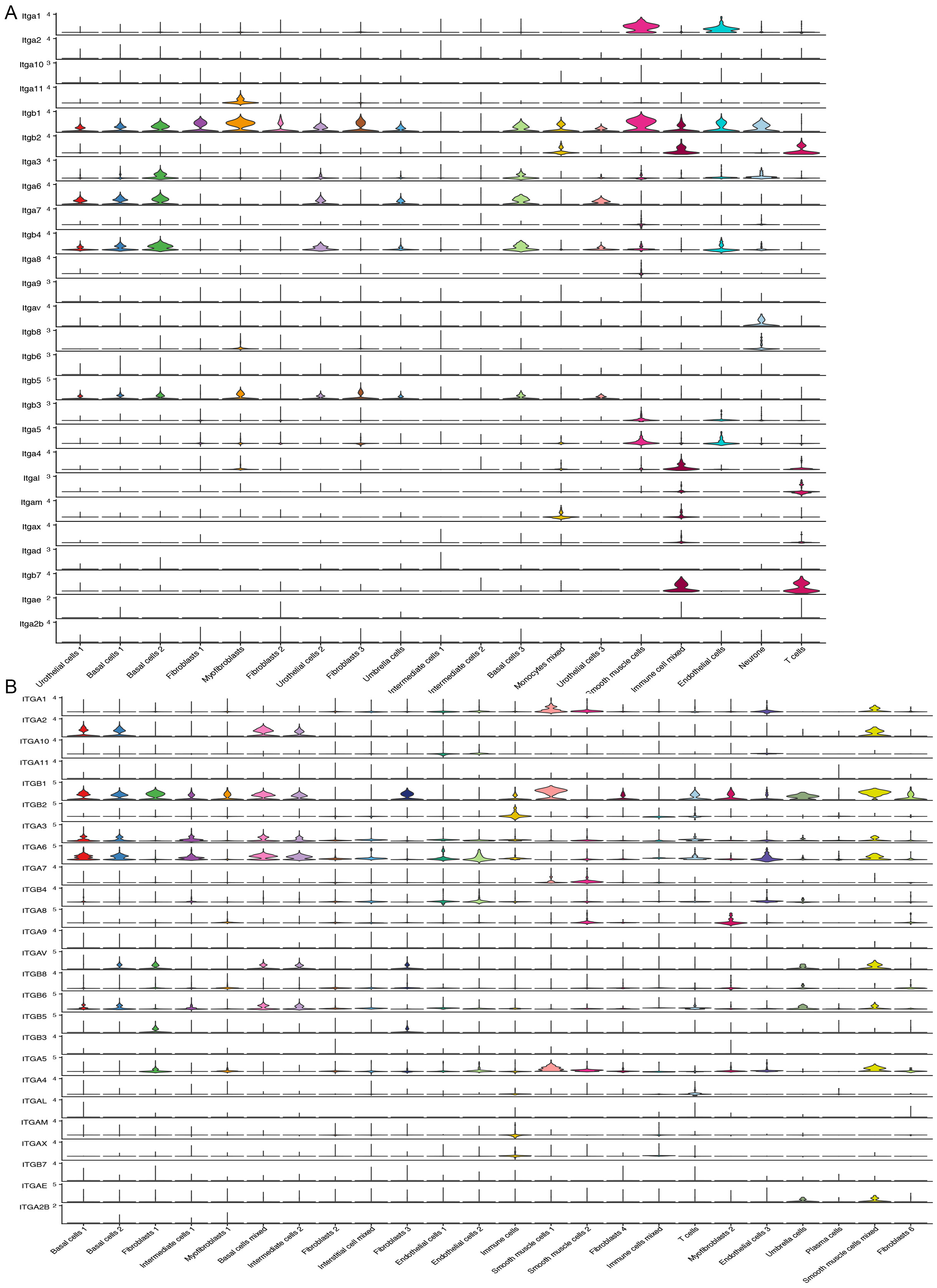

Supplement: Supplementary file 7 — Fig S7 [file CPR-55-e13170-s008.jpg]

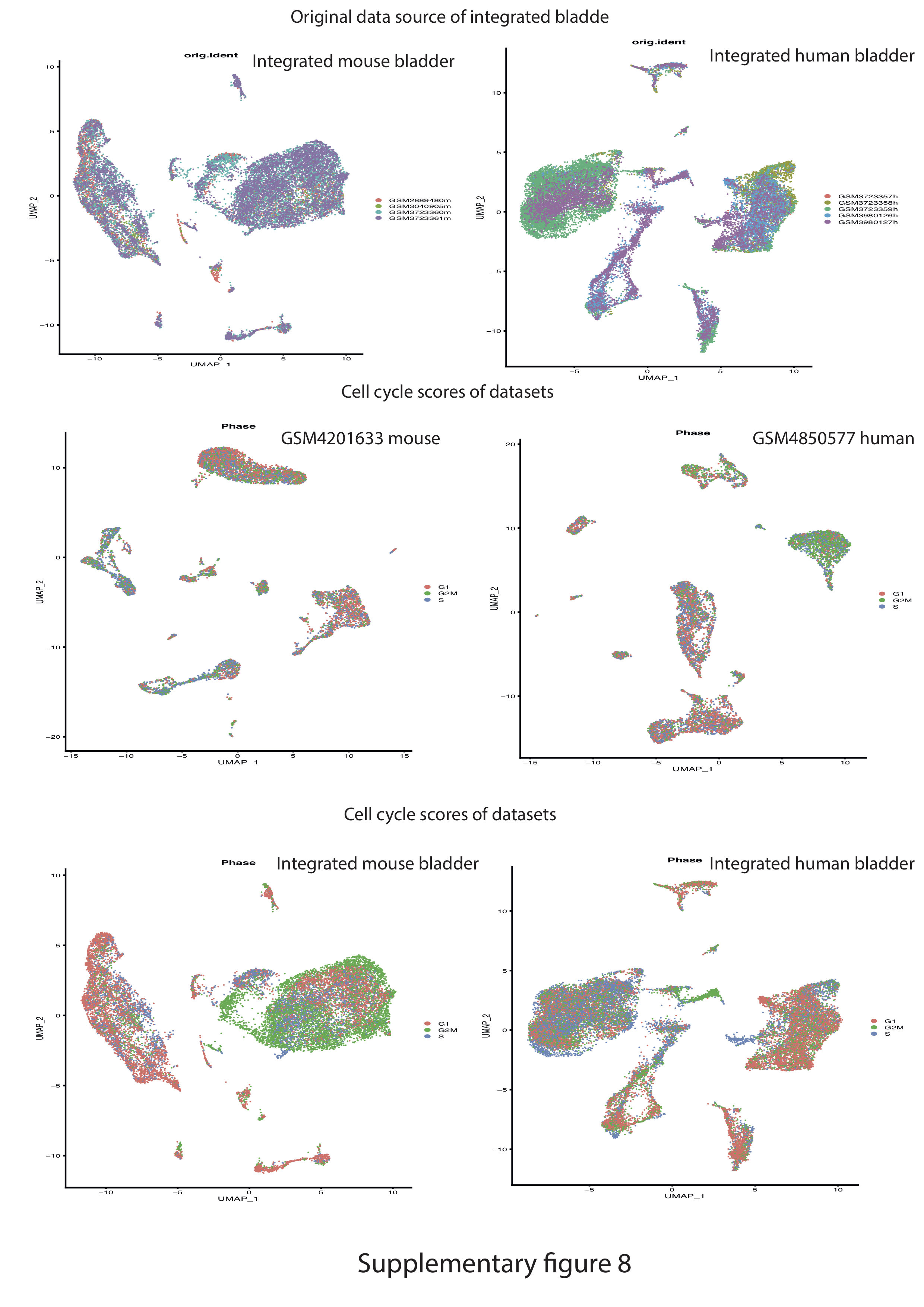

Supplement: Supplementary file 8 — Fig S8 [file CPR-55-e13170-s005.jpg]
